# atBioNet– an integrated network analysis tool for genomics and biomarker discovery

**DOI:** 10.1186/1471-2164-13-325

**Published:** 2012-07-20

**Authors:** Yijun Ding, Minjun Chen, Zhichao Liu, Don Ding, Yanbin Ye, Min Zhang, Reagan Kelly, Li Guo, Zhenqiang Su, Stephen C Harris, Feng Qian, Weigong Ge, Hong Fang, Xiaowei Xu, Weida Tong

**Affiliations:** 1 ICF International at FDA's National Center for Toxicological Research, 3900 NCTR Rd, Jefferson, AR, 72079, USA; 2 Divisions of Bioinformatics and Biostatistics, National Center for Toxicological Research, US Food and Drug Administration, 3900 NCTR Road, Jefferson, AR, 72079, USA; 3 Department of Lymphoma and Myeloma, University of Texas M D Anderson Cancer Center, Houston, TX, 77054, USA; 4 State Key Laboratory of Multiphase Complex Systems, Institute of Process Engineering, Chinese Academy of Sciences, Beijing, 100190, P. R. China; 5 Department of Information Science, University of Arkansas at Little Rock, 2801 S. University Ave., Little Rock, AR, 72204-1099, USA

**Keywords:** Protein-protein interaction, Network analysis, Functional module, Disease biomarker, KEGG pathway analysis, Visualization tool, Genomics

## Abstract

**Background:**

Large amounts of mammalian protein-protein interaction (PPI) data have been generated and are available for public use. From a systems biology perspective, Proteins/genes interactions encode the key mechanisms distinguishing disease and health, and such mechanisms can be uncovered through network analysis. An effective network analysis tool should integrate different content-specific PPI databases into a comprehensive network format with a user-friendly platform to identify key functional modules/pathways and the underlying mechanisms of disease and toxicity.

**Results:**

atBioNet integrates seven publicly available PPI databases into a network-specific knowledge base. Knowledge expansion is achieved by expanding a user supplied proteins/genes list with interactions from its integrated PPI network. The statistically significant functional modules are determined by applying a fast network-clustering algorithm (SCAN: a Structural Clustering Algorithm for Networks). The functional modules can be visualized either separately or together in the context of the whole network. Integration of pathway information enables enrichment analysis and assessment of the biological function of modules. Three case studies are presented using publicly available disease gene signatures as a basis to discover new biomarkers for acute leukemia, systemic lupus erythematosus, and breast cancer. The results demonstrated that atBioNet can not only identify functional modules and pathways related to the studied diseases, but this information can also be used to hypothesize novel biomarkers for future analysis.

**Conclusion:**

atBioNet is a free web-based network analysis tool that provides a systematic insight into proteins/genes interactions through examining significant functional modules. The identified functional modules are useful for determining underlying mechanisms of disease and biomarker discovery. It can be accessed at: http://www.fda.gov/ScienceResearch/BioinformaticsTools/ucm285284.htm.

## Background

Protein-protein interaction (PPI) networks and other network biology techniques have been widely used to study human disease [1-3]. It is believed that perturbations in cellular networks might provide insight into the mechanisms underlying diseases. PPI network analysis, either alone or combined with other information, has been successfully applied in identifying disease associated biomarkers and pathways [2]. Network-based classification has also shown promise in cancer diagnosis and prognosis. Network-based biomarkers have not only successfully been used for classification of metastatic versus non-metastatic tumors, but also demonstrated higher reproducibility compared to individual marker genes identified by conventional approaches [4].

Omics techniques utilizing, for example, gene expression data from microarrays or next-generation sequencing as well as proteomic or metabolomic data have become a standard practice to identify candidate molecular biomarkers. The common way to interpret and contextualize these biomarkers is with enrichment analysis using Gene Ontology [5], Kyoto Encyclopedia of Genes and Genomes (KEGG) [6] and other similar approaches. This type of analysis emphasizes the functional relationship of markers. Alternatively, the omics data can be interrogated based on their inherent connection and association in a network form.

Network-based analysis represents an advanced systems biology methodology to understand and interpret the complex omics data [7]. By considering the cross-talking of multiple pathways, network modeling allows a more comprehensive analysis of a complex system than the pathway-centric approach. Additionally, the unsupervised nature of network analysis provides opportunities for identifying novel relationships not captured in the pre-defined pathways, and thus offers a distinct approach for biomarker discovery [8,9]. It has been shown that network topological properties can be used for prioritizing candidate disease genes and predicting novel candidate biomarkers [10], and modularity analysis could extract relevant sub-networks related to the studied disease [11]. Therefore, network-based analysis has played an increasing role in modern biomarker discovery and drug development. For example, using network-based analysis, insulin signaling and nuclear receptor networks were found consistently to be differentially expressed in many type 2 diabetes models of insulin resistance [4], and a core network underlying the insulin signaling pathway impaired in patients who are insulin resistant was also identified [12].

A number of software programs have been developed for network analysis and visualizations; a comprehensive list was compiled by Gehlenborg et al. [13]. Some programs focus on the graphical visualization of the network [14-19], while others also add computational functions such as cluster analysis [20-23] and modularity identification [24], aiding in the interpretation of the biological functions underlying the complex networks. Cytoscape [15], MATISSE [9], VisANT [25], PINA [2] and Gene2Networks [11] are among a few representing the endeavor that has been made in this field.

We developed atBioNet, a free web-based tool for genomic and proteomic data, that can perform network analysis followed by biological interpretation for a list of seed proteins/genes (i.e., proteins/genes provided by user). The distinct advantages of atBioNet over other existing systems are that: (1) it is an integrated system, where all the key steps in network analysis are combined into a user-friendly interface; (2) atBioNet can identify new functionally related proteins and genes in the context of a PPI network built from seven popular public databases; (3) atBioNet provides a fast network-clustering algorithm called Structural Clustering Algorithm for Networks (SCAN) to identify functional modules; and (4) KEGG pathway information has been seamlessly connected to the atBioNet interface for the assessment of biological functions of the modules through enrichment analysis. Here we present details of the atBioNet application and provide the analyses of three example disease cases (acute leukemia, systemic lupus erythematosus, and breast cancer) to illustrate its utility in real-world applications.

## Implementation

atBioNet was developed at the U.S. Food and Drug Administration’s National Center for Toxicological Research (NCTR). It can be accessed at: http://www.fda.gov/ScienceResearch/BioinformaticsTools/ucm285284.htm.

The application takes a list of proteins/genes and places them in a PPI network to identify functional modules through SCAN and enrichment analysis. For each module, the seed proteins/genes are highlighted. Other proteins/genes in the same module are expected to share similar functions as the seed proteins/genes and thus could be novel biomarkers for the disease or toxicity associated with the seed proteins/genes. A wide variety of protein ID or gene ID formats are supported including Entrez Gene ID, GenBank accession, official gene name, and many more.

### PPI database

The atBioNet contains a built-in PPI database integrating seven public PPI databases, which includes BioGRID [26], The DIP^TM^[27], HPRD [28], IntAct [29], MINT [30], REACTOME [31], and SPIKE [32]. The detailed information for the seven databases is listed in Table 1.

**Table 1:** Information for the seven public PPI databases

**Databases**	**Description**	**Extracted*Homo sapien*proteins and interactions**	
		**Number of proteins**	**Number of interactions**
BioGRID thebiogrid.org	BioGRID provides PPI data compiled through comprehensive curation efforts from high-throughput data sets and individual focused studies.	8204	33625
DIP dip.doembi.ucla.edu/dip/Main.cgi	The DIP^TM^ catalogs experimentally determined interactions between proteins, mainly from yeast, and includes interactions from Helicobacter pylori and human.	1137	1509
HPRD http://www.hprd.org	The HPRD provides submitted human PPI data including mass spectrometry and protein microarray-derived data among other data types.	9553	38802
IntAct http://www.ebi.ac.uk/intact/main.xhtml	IntAct contains PPI data with full descriptions of the experimental conditions; data is derived from literature curation or direct user submissions.	7495	30965
MINT mint.bio.uniroma2.it/mint/Welcome.do	MINT focuses on experimentally verified PPIs classified as human, domain–peptide, and virus–virus/host. Data is mined from the scientific literature by expert curators.	5230	15353
REACTOME http://www.reactome.org	REACTOME collects manually curated and peer-reviewed pathway data for all species.	3599	74490
SPIKE http://www.cs.tau.ac.il/~spike/	SPIKE focuses on highly curated human signaling pathways.	6927	23224

The disparate protein IDs in different databases were consolidated using the Entrez Gene ID, from which the seven databases were combined. There are two database options to choose from in our application, corresponding to two different approaches of combining the seven databases. The default option is “Human Database” that took a union of human proteins from the seven databases. This database consists of 12043 human proteins and 132605 interactions. A more stringent option “K2 Human Subset Database” only considers a smaller and more robust database with 9104 proteins and 36088 interactions obtained by the integration of the seven original databases using the *k*-votes approach, presented in our previous publication, with *k* = 2 indicating that PPIs must appear in at least two of the seven original databases [33].

### PPI network

A PPI network is a collection of nodes (i.e., proteins/genes) and edges (interactions). There are several ways to generate a PPI network in atBioNet. By default, the network is created by adding proteins/genes from the PPI database that directly interact with the seed proteins/genes when the number of input nodes is less than 1000. Edges are added in the network only for pairs of nodes where at least one node represents a seed proteins/genes. However, when a user begins with a large number of input proteins/genes, more stringent options can be used, such as including only proteins/genes that connect to more than two seed proteins/genes, or using only input nodes. Currently, “use only input nodes” is the default option when the number of input proteins/genes is greater than 1000. The aforementioned options are provided in atBioNet so that the user can select the scope of the generated PPI network.

### Functional modules

Once the PPI network is established, atBioNet provides an on-the-fly network algorithm to analyze the network. The algorithm is based on SCAN, which identifies statistically significant clusters or functional modules based on the structural similarity of a pair of vertices connected by an edge [34]. Structural similarity is calculated based on their common neighbors. The algorithm aims to assign a vertex to a cluster where it shares many common neighbors with other members of the cluster. SCAN runs linearly in terms of the size of the network, which allows the user to analyze large networks with a much shorter time in comparison with most other algorithms. Another key feature of SCAN is the identification of nodes with special roles in the network such as hubs and outliers. Hubs are nodes that bridge different modules, thus the hub proteins/genes could play multiple roles related to the mechanisms represented in the connected modules. Outliers are nodes that have weak or no connection to all the modules, and thus the outlier proteins/genes may hold a distinct role in biology. Various statistical network measures can be calculated, including Page Rank, Degree Centrality, HITS, and BETWEENNESS. These measures can be exported in several formats such as tab delimited and GUESS’s GDF format [35].

### atBioNet visualization

The graphical network in atBioNet is generated using GUESS, an open source network visualization and exploration tool (provided by Eytan Adar at the University of Michigan) [35]. The network layout algorithm used is a Generalized Expectation-Maximization (GEM) algorithm described by Arne et al. [36].

**Figure 1:**
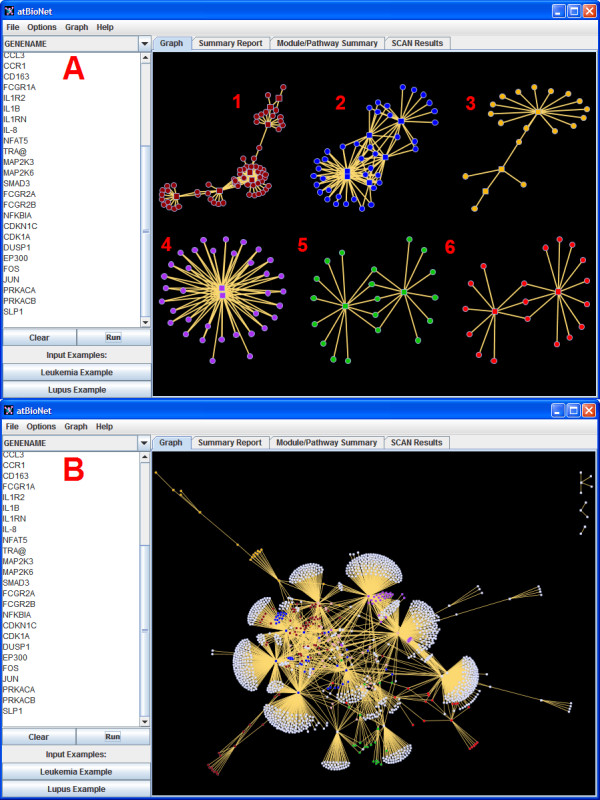
**atBioNet interface.** The network visualization for the systemic lupus erythematosus data in atBioNet’s interface for both the top 6 modules (**A**) and the entire network (**B**). Square nodes represent seed proteins/genes and circles are added by the network.

The interface of atBioNet is shown in Figure 1. The default setting is to display the six top modules as separate entities (Figure 1A), allowing the user to focus on the most significant modules of the network. Depending on the user’s goals, the modules can be ranked either by the number of seed proteins/genes, or the total number of proteins/genes in the module, or Mark Newman’s modularity score [37]. Mark Newman’s modularity score is originally defined as a quality measure of the whole clustering. We generalized Mark Newman’s modularity for each module as a quality measure. For a complete view, the entire network (the largest 6 modules retain their coloring) can be shown as well (Figure 1B).

### Pathway analysis

The KEGG pathway database [38] has been integrated into atBioNet to support further data interpretation. The number of pathways for each network module created from the seed proteins/genes is listed within atBioNet. A pathway summary ranked by Fisher’s exact test p-value showing the relevant seed proteins/genes and category for each KEGG pathway is available for each individual module. In addition, the identified pathways are directly linked to its detailed view on the KEGG website highlighting the present proteins/genes within the module.

## Results

**Figure 2:**
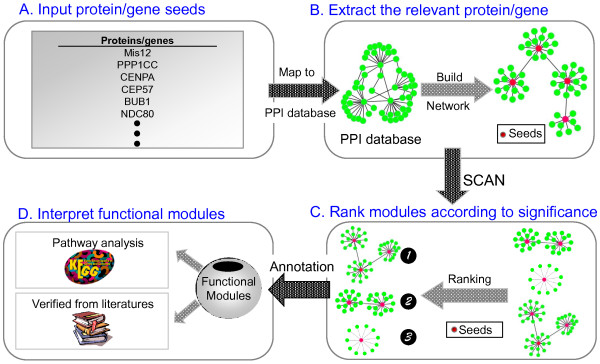
**atBioNet workflow.** Flowchart of an example use case of atBioNet. The user inputs a Proteins/genes list (**A**); a network is created (**B**); ranked in order of significance (**C**); and then the results are interpreted for their biological significance (**D**).

The flowchart in Figure 2 depicts one common workflow using atBioNet for data analysis. First, a list of proteins/genes that the user is interested in is inputted into atBioNet as the network seeds. Then, the database is searched for other proteins/genes known to directly interact with the network seeds, and the network is built. The clustering algorithm SCAN is used to identify functional modules based on the network structural similarity, and then these modules are ranked according to their significance, i.e., the number of seed nodes, total number of nodes, or modularity score. Finally, the data presented in the network can be used for various applications such as finding associated pathways, validating current literature findings, and discovering new biomarkers.

We present three case studies below to demonstrate the utility of atBioNet in clinical applications: the differentiation of acute myeloid leukemia from acute lymphoblastic leukemia [39], diagnosis of systemic lupus erythematosus [40], and prognosis of breast cancer [41]. The initial publication for each of these studies provided a list of genes (biomarkers), which were inputted into atBioNet (see Additional file 1). The gene count summary for three case studies is shown in Table 2, and the top 2 modules and top 10 pathways per module are shown in Table 3.

**Table 2:** Summary of the gene counts from the three case studies

**Study case [reference]**	**Acute leukemia [**[39]**]**	**Lupus [**[40]**]**	**Breast cancer [**[41]**]**
Original published genes	50	37	70
# inputted as seed genes in atBioNet	46	37	65
Mapped genes from atBioNet	44	37	50
Added genes from atBioNet	1362	1312	856
Added edges from atBioNet	1671	2160	1089

**Table 3:** Top 10 KEGG pathways ranked by p-value for the top two modules in the three disease case studies

**Disease**	**Functional Modules (# gene)**	**Map Title in KEGG**	**# of genes mapped in the pathway**	**Fisher P value**
Acute leukemias	Module #1: Leukemia module (n = 44)	Huntington's disease(hsa05016)	6	<0.0001
		Cell cycle(hsa04110)	5	0.00015
		Chronic myeloid leukemia(hsa05220)	4	0.00021
		Prostate cancer(hsa05215)	4	0.00045
		Notch signaling pathway(hsa04330)	3	0.00092
		Pathways in cancer(hsa05200)	6	0.00184
		Measles(hsa05162)	4	0.00209
		Neuroactive ligand-receptor interaction(hsa04080)	5	0.00465
		Primary immunodeficiency(hsa05340)	2	0.00913
		Endometrial cancer(hsa05213)	2	0.01948
	Module #2: Immune module (n = 32)	JAK-STAT signaling pathway(hsa04630)	12	<0.0001
		B cell receptor signaling pathway(hsa04662)	8	<0.0001
		Primary immunodeficiency(hsa05340)	8	<0.0001
		Measles(hsa05162)	7	<0.0001
		Natural killer cell mediated cytotoxicity(hsa04650)	7	<0.0001
		Osteoclast differentiation(hsa04380)	6	<0.0001
		Hematopoietic cell lineage(hsa04640)	5	<0.0001
		T cell receptor signaling pathway(hsa04660)	5	0.00012
		Chemokine signaling pathway(hsa04062)	6	0.00019
		Chronic myeloid leukemia(hsa05220)	4	0.00033
Lupus	Module #1: Inflammatory Module (n = 69)	MAPK signaling pathway(hsa04010)	28	<0.0001
		Cell cycle(hsa04110)	12	<0.0001
		Osteoclast differentiation(hsa04380)	15	<0.0001
		Toll-like receptor signaling pathway(hsa04620)	16	<0.0001
		NOD-like receptor signaling pathway(hsa04621)	14	<0.0001
		GnRH signaling pathway(hsa04912)	12	<0.0001
		Pertussis(hsa05133)	12	<0.0001
		Leishmaniasis(hsa05140)	10	<0.0001
		Chagas disease (American trypanosomiasis)(hsa05142)	11	<0.0001
		Toxoplasmosis(hsa05145)	13	<0.0001
	Module #2: Immune module (n = 49)	Osteoclast differentiation(hsa04380)	13	<0.0001
		JAK-STAT signaling pathway(hsa04630)	12	<0.0001
		Measles(hsa05162)	10	<0.0001
		Influenza A(hsa05164)	12	<0.0001
		Pathways in cancer(hsa05200)	13	<0.0001
		Hepatitis C(hsa05160)	8	<0.0001
		Leishmaniasis(hsa05140)	5	<0.0001
		Basal transcription factors(hsa03022)	4	0.00025
		Toll-like receptor signaling pathway(hsa04620)	5	0.00028
		Acute myeloid leukemia(hsa05221)	4	0.00033
Breast cancer	Module #1: Proliferative module (n = 192)	DNA replication(hsa03030)	16	<0.0001
		Nucleotide excision repair(hsa03420)	13	<0.0001
		ErbB signaling pathway(hsa04012)	15	<0.0001
		Cell cycle(hsa04110)	41	<0.0001
		Pathways in cancer(hsa05200)	28	<0.0001
		Renal cell carcinoma(hsa05211)	13	<0.0001
		Pancreatic cancer(hsa05212)	13	<0.0001
		Chronic myeloid leukemia(hsa05220)	16	<0.0001
		Focal adhesion(hsa04510)	20	<0.0001
		Measles(hsa05162)	14	<0.0001
	Module #2: Metastasis module (n = 74)	Focal adhesion(hsa04510)	17	<0.0001
		ECM-receptor interaction(hsa04512)	15	<0.0001
		Amoebiasis(hsa05146)	11	<0.0001
		Pathways in cancer(hsa05200)	14	<0.0001
		Protein digestion and absorption(hsa04974)	8	<0.0001
		Small cell lung cancer(hsa05222)	7	<0.0001
		Bladder cancer(hsa05219)	4	0.00019
		Malaria(hsa05144)	4	0.00041
		Rheumatoid arthritis(hsa05323)	5	0.00041
		Cytokine-cytokine receptor interaction(hsa04060)	8	0.00042

### Case study 1: differentiation of acute myeloid leukemia (AML) from acute lymphoblastic leukemia (ALL)

Acute leukemia is a cancer of the blood cells, with two predominant forms known as ALL (acute lymphoblastic leukemia, arising from lymphoid precursors) and AML (acute myeloid leukemia, arising from myeloid precursor) [39]. Distinguishing ALL from AML is critical for successful treatment, since the chemotherapy regimens for ALL are different from those for AML [39]. By decreasing the misdiagnosis rate of AML and ALL, unwarranted toxicities will be reduced and cure rates will be increased.

A signature with 50 genes to distinguish AML and ALL were identified and published by Golub et al. [39]. Forty-six genes were matched using GenBank from the National Center for Biotechnology Information (NCBI) based on the gene name provided. They were inputted into atBioNet as seeds to generate significant modules. Two distinct modules were identified (Table 3).

In module 1, top ten KEGG pathways were listed (Table 3), most of them related to cancer development and progression. The chronic myeloid leukemia pathway was identified in this module, implying that module 1 is AML-specific. For example, four genes (i.e., GRB2, HDAC1, HDAC2, and TP53) identified in the chronic myeloid leukemia pathway are known to be distinguish AML from ALL, indicating that other genes in this module might also be potential biomarkers for AML.

Immune response is one of major factors influencing etiology of acute leukemia [42]. Many genes in the second module are involved in the function of the immune system; the enriched pathways in this module were also associated with the immune system, including primary immunodeficiency, natural killer cell mediated cytotoxicity, T cell receptor signaling pathway, and chemokine signaling pathway.

### Case study 2: diagnosis of systemic lupus erythematosus (SLE)

SLE is a chronic inflammatory autoimmune disease in which antibodies attack self-antigens leading to damage in many organ systems, including the bones, joints, kidneys, and central nervous system. Inflammation and the production of auto-antibodies play an important role in the pathogenesis of SLE [43].

A 37-gene meta-signature biomarker panel for SLE [40] was used as the seed genes in atBioNet, and the resulting top two modules are shown in Table 3. The first module was related to inflammatory processes. In this module, 28 of 69 genes were involved in the MAPK signaling pathway, which regulates the synthesis of inflammatory mediators at the level of transcription and translation [44]. Genes such as IL1B, TLR3, and TICAM1 from the Toll-like receptor signaling pathway and CASP1, IL1B from the NOD-like receptor signaling pathway, which are vital for generating mature pro-inflammatory cytokines, were also identified in this module [45,46].

The second module was related to immune activity. It included osteoclastogenesis, which is mainly regulated by signaling pathways activated by immune receptors. The JAK–STAT, which is a signaling pathway with an important role in the control of immune responses, was also implicated. Dysregulation of the JAK-STAT pathway is associated with various immune disorders; because biomarkers may not be unique to a specific disease, they are good candidates for further investigation [47].

**Figure 3:**
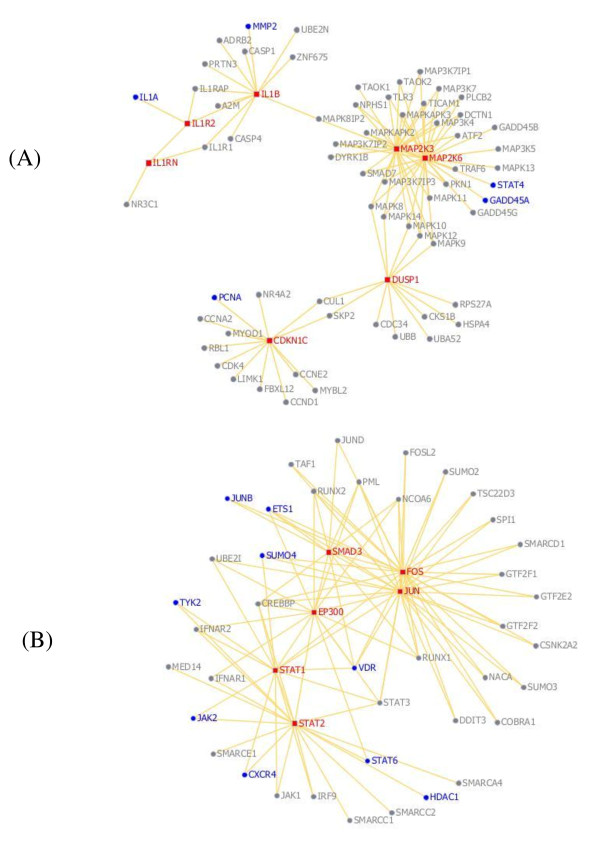
**Known and potential SLE biomarkers found by atBioNet.** Additional SLE biomarker genes found based on the 37 seed genes using atBioNet. Module 1 (**A**) and module 2 (**B**) are shown. The red squares represent the seed genes, and the light blue circles represent the identified SLE biomarker genes that are confirmed by literatures.

A total of 14 genes, five in the first module and nine in the second module (highlighted in Figure 3), have previously been identified in the literature as possible biomarkers for SLE. For example, deletion of the Gadd45a gene (Figure 3A) in mice is associated with the development of an autoimmune disease similar to human SLE, suggesting this gene plays a vital role in SLE development [48]. Similarly, variants of many genes found in the second module (Figure 3B), including ETS1, STAT6, VDR, and TYK2, were found to be associated with SLE [49-53]. Details for the 14 literature-confirmed potential SLE biomarkers are listed in Additional file 2.

### Case study 3: prognosis of breast cancer

Breast cancer is the most common malignant disease in Western women. Adjuvant chemotherapy has made a significant contribution to the improvement of breast cancer survival rates. However, considering the toxic effects and high cost associated with chemotherapy, developing better prognostic biomarkers that identify which breast cancer patients do not need additional chemotherapy is still a pressing clinical challenge for the management of breast cancer patients [54].

The 70-gene signature used in MammaPrint® for breast cancer prognosis was analyzed, and 65 of these genes were found in GenBank. These genes were imported into atBioNet to create a breast cancer prognosis related network, and several modules were identified. The top module shown in Table 3 was a cancer cell proliferation module. Most of the pathways in the first module were related to the proliferation of cancer cells, e.g., DNA replication, nucleotide excision repair, ErbB signaling pathway, and cell cycle regulation.

The second module shown in Table 3 reflects the invasive aspect related to cancer metastasis. The first two pathways (i.e., focal adhesion and ECM-receptor interaction) reflect the invasive capacity of the tumor cell to escape from their primary site. These two pathways could not be statistically enriched by the seed genes, providing additional evidence that atBioNet can identify novel mechanisms related to the studied disease.

## Discussion

We reported a user-friendly network analysis and interpretation tool called atBioNet and described three case studies using atBioNet to identify key functional modules and provide hypotheses for the underlying mechanisms of diseases based on proteins/genes lists comprising candidate biomarkers from omics technologies. atBioNet leverages existing knowledge from seven publicly available PPI databases and adds powerful network analysis and visualization tools. The system has the capability to expand knowledge based on a list of seed proteins/genes through analysis of the resulting functional modules. The functional modules were identified by using SCAN, a fast structural clustering method, and annotated with KEGG pathways.

Recent advances in omics technologies have generated huge amounts of publicly available PPI data. Several visualization and network analysis tools have been developed to leverage this data for different purposes. VisANT [55] is an integrative framework for the analysis, mining, and visualization of pathways and integrated omics data. VisANT generates networks for use in systems biology research from input proteins/genes by querying integrated PPI data from multiple sources[56]. The resulting network is annotated by using information from KEGG[57] and GO[58]. PINA [2] is another network construction, analysis, and visualization tool that contains information from six public PPI databases. It contains ~2400 pre-determined modules. Given a input proteins/genes, PINA determined the over-expressed modules by performing an enrichment test and then offer biological context to the modules that are annotated with GO, KEGG, protein domains, and MsigDB [59]. Unlike PINA, atBioNet constructs modules at the time of the query, which is dynamic and allows novel modules to be generated based on the input proteins/genes. NAViGaTOR [19] mainly focuses on 2D or 3D visualization of PPI networks as well as GO annotation of the nodes. Cytoscape [60] allows users to build a customized pipeline to analyze PPI data by using different plug-ins and annotation tools, but the effective use of Cytoscape requires a thorough understanding of the tools and plug-ins available and expertise in organizing and interpreting the output.

atBioNet performs functional module analysis and biomarker identification by integrating public PPI data sources. atBioNet begins from the hypothesis that proteins/genes in the same module are likely involved in the same biological functions or processes. This approach allows un-annotated proteins/genes to be used as potential biomarkers for the same human disease that the input proteins/genes are associated with. Furthermore, sub networks are detected using the SCAN algorithm [34], which has been demonstrated to be a powerful tool for large-scale network analysis from both statistical and biological points of views.

More specifically, the SCAN algorithm quickly, efficiently, and accurately analyzes networks. SCAN’s runtime scales linearly with the size of the network, which makes it a scalable approach for extremely large networks with hundreds of thousands or even millions of nodes. Moreover, SCAN accurately finds clusters, and also identifies nodes playing crucial roles with only one traverse of the network. The power of SCAN has been demonstrated in applications including PPI networks [33,61] and social networks [62,63] in addition to the three study cases we have examined in this study.

From a clinical point of view, the rationale behind the functional module analysis and biomarker discovery performed by atBioNet is to find effective and robust biomarkers for a disease. When the number of candidate genes is too small to identify functional module, additional proteins/genes can be added from atBioNet's database to expand the network. In contrast, when there is a large amount of input proteins/genes associated with a phenotype, atBioNet focuses on detecting functional modules, the hub genes (e.g., transcription factors or regulatory genes), and outlier genes based solely on the list of seed proteins/genes. Thus, potential biomarkers that are important to multiple biological processes, mechanisms, or functions can be identified.

The three case studies presented here each used the default network parameters and the results were consistent with the knowledge about these diseases. atBioNet provides several options for network analysis, such as the choice of the starting PPI database, control of the stringency of node additions during network construction, etc. The particular options used will depend on specific research questions and scenarios; for example, for a very large list of seed proteins/genes, the user may choose to construct a network using only the seed proteins/genes without adding any additional nodes. To build a more reliable network, the user can choose to use a smaller, more stringent database [33].

Moreover, all three case studies are based on single genomic signature as a seed for network analysis. Actually, the network approach will be more powerful by using multiple signatures reported in different literature studies for a particular disease to enhance the accuracy of the functional modules interpreting the underlying mechanisms of the disease. It has been well-documented that different studies of the same disease often produce gene signatures with few overlapping elements [64], but they might reflect different mechanisms associated with the disease. Using atBioNet, different signatures can be integrated into the genome-wide network view, which can be used to further our understanding of biomarker specificity and broadening the search space and thus potentially offering a more comprehensive view of the PPI networks underlying the disease.

Another potential use of atBioNet is to study the mechanisms related to therapeutic use of drug combinations, which have become very effective due to medicinal research advancements in recent years [65]. We can combine the signature genes associated with each drug and use the union list as a seed for network analysis. While individual drugs may affect a set of regulatory genes or pathways, combining drug actions in the context of biological mechanisms underlying the disease condition could lead to more effective therapies for a complicated clinical situation.

In the current version, atBioNet contains only human protein-protein interactions. Our next major revision will expand the available data to include the STRING and NCBI PID databases as well as covering PPI data from other species. Another limitation of the current atBioNet is that the biological annotation is exclusively relied on KEGG. We will add other biological annotation sources in the future such as GO, Biocarta pathways, disease-centric databases, and more. Additionally, owing to memory constraints in Java, there is an upper limit of approximately 3000 seed proteins/genes when using the “add all directly connected nodes” option in atBioNet. Nevertheless, the user is able to allocate more memory to the application to allow network analysis for a larger number of seed proteins/genes.

## Conclusions

We implemented atBioNet as a web-based tool that provides a convenient platform for human-specific network analysis with a focus on identifying biologically relevant functional modules. The three case studies presented here demonstrate the utility of atBioNet in discovering biomarkers and mechanisms in human diseases. The power of integrating the SCAN algorithm, custom PPI database, visualization, and user friendly interface to allows atBioNet users to build biologically meaningful interpretations of the relationships among the proteins/genes implicated in the constructed networks. Finally, atBioNet will undergo continual development and will potentially be expanded to handle omics data and systems biology studies.

## Availability and requirements

Project name: atBioNet.

Project home page: http://www.fda.gov/ScienceResearch/BioinformaticsTools/ucm285284.htm.

Operating system(s): Platform independent; tested on Windows XP/Vista/7, Linux/Ubuntu/Redhat, and Mac (with an Intel core2 duo or better).

Programming language: Java.

Other requirements: Java 1.6 or higher, 1 GB RAM.

License: None required.

Any restrictions to use by non-academics: No.

## Abbreviations

ALL, Acute lymphoblastic leukemia; AML, Acute myeloid leukemia; GO, Gene ontology; GEM, Generalized expectation-maximization; KEGG, Kyoto encyclopedia of genes and genomes; NCTR, National center for toxicological research; NCBI, National center for biotechnology information; PPI, Protein-protein interaction; SLE, Systemic lupus erythematosus; SCAN, Structural clustering algorithm for networks.

## Competing interests

The authors declare that they have no competing interests.

## Authors’ contributions

YD developed atBioNet tool. MC contributed to case studies analysis. WT conceived the original idea and methods, and HF guided development. DD, ZL, MC, MZ, HF, YD, and XX wrote the first draft. YY, ZS, SH, and LG contributed to the construction of the PPI databases and networks. ZL, FQ, WG, DD, RK and HF contributed to testing and improving software. WT improved the manuscript. All authors read and approved the final manuscript.

## Disclaimer

The views presented in this article do not necessarily reflect those of the US Food and Drug Administration.

## Supplementary Material

Additional file 1 **Description of data: List of all seed genes using various IDs in all three case studies.** The highlighted columns are the input gene ID at atBioNet. Click here for file

Additional file 2 The 14 literature-identified potential SLE biomarkers in case study 2. Click here for file
